# Stacking triple genes increased proanthocyanidins level in *Arabidopsis thaliana*

**DOI:** 10.1371/journal.pone.0234799

**Published:** 2020-06-25

**Authors:** Jiebing Wei, Junfeng Yang, Wenbo Jiang, Yongzhen Pang

**Affiliations:** 1 Key Laboratory of Plant Resources and Beijing Botanical Garden, Institute of Botany, Chinese Academy of Sciences, Beijing, China; 2 Institute of Animal Science, Chinese Academy of Agricultural Sciences, Beijing, China; 3 University of Chinese Academy of Sciences, Beijing, China; Indiana University, UNITED STATES

## Abstract

Anthocyanins and proanthocyanidins are two important plant secondary metabolites, and they contribute to plant survival and human health. In particular, proanthocyanidins could also prevent ruminants from the damage of pasture bloat. However, the improvement of proanthocyanidins content remain unsatisfied. In this study, we attempted to improve proanthocyanidins level by gene stacking in *Arabidopsis thaliana* as prove-of-concept. Two proanthocyanidin pathway genes from tea plant, *CsF3’5’H* and *CsANR2*, were co-expressed in the wild type and *PAP1* over-expression *Arabidopsis*. Over-expression of *CsF3’5’H* slightly affected anthocyanins level in leaves and proanthocyanidins in mature seed when expressed alone in the *pap1-D* line. Over-expression of *CsANR2* led to an obvious decrease in anthocyanins in leaves of both wild type and *pap1-D* lines, but increase in proanthocyanidin level in mature seeds. Over-expression of *CsANR2* in *pap1-D* lines lead to production of DMACA-reactive soluble proanthocyanidins in leaves, but not in wild type or *pap1-D l*ines. Anthocyanins level was decreased in the leaves of *CsF3’5’H*, *CsANR2* and *pap1-D* co-expression lines, but proanthocyanidins were increased remarkably in both leaves and mature seeds in the co-expression line. It is concluded that co-expression of *CsANR2* and *PAP1* in *Arabidopsis* produce soluble proanthocyanidins in leaves, and co-expression of *CsF3’5’H*, *CsANR2* and *PAP1* lead to a significant increase in proanthocyanidins in mature seeds. The transcript levels of endogenous *CHS*, *DFR*, *ANS* and *ANR* genes in *Arabidopsis* were up-regulated in the triple genes co-expression line. Based on these studies, it is possible to develop new plant germplasm with improved proanthocyanidins by co-expressing of multiple genes.

## Introduction

Flavonoids are one of the most important secondary metabolite groups in plant, among them, anthocyanins and proanthocyanidins are two important branch products in flavonoid pathway. Anthocyanins contribute to plant coloration and survival [1], and proanthocyanidins provide defense against plant pathogens and other diseases [2,3]. Both anthocyanins and proanthocyanidins showed numerous beneficial effects on human health [4,5]. For animal health, it has been suggested that modest level of proanthocyanidins in the leaves and stems of alfalfa and white clover could prevent pasture bloat which is lethal in ruminant animals [6].

**Fig 1:**
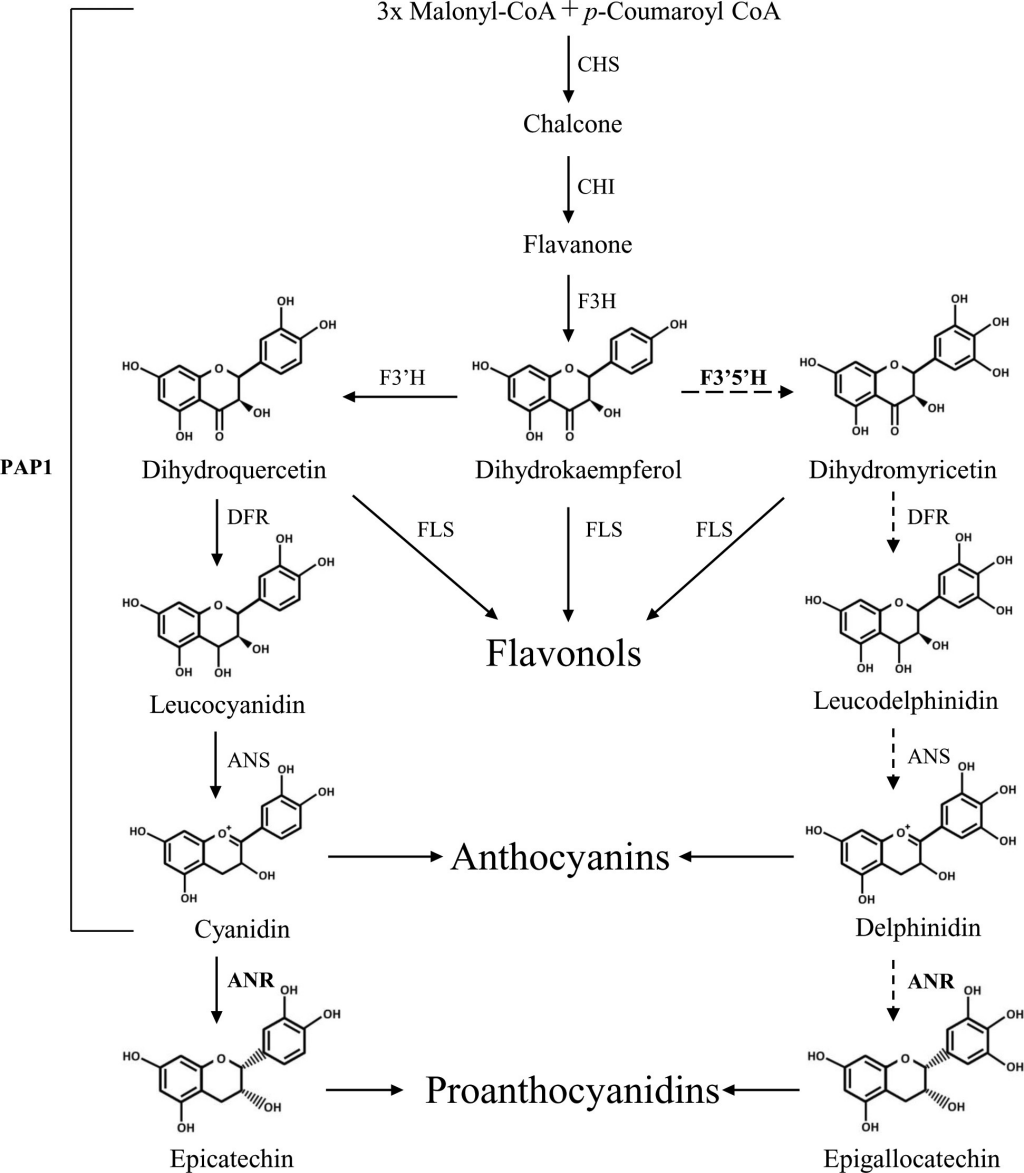
General flavonoid biosynthetic pathway in *Arabidopsis*. Abbreviations: ANR, anthocyanidin reductase; ANS, anthocyanidin synthase; CHI, chalcone isomerase; CHS, chalcone synthase; DFR, dihydroflavonol 4-reductase; F3H, flavanone 3-hydroxylase; F3′H, flavanone 3′-hydroxylase; FLS, flavonol synthase, F3′5′H, flavonoid 3′,5′-hydroxylase, dashed lines indicated the proposed pathway.

In *Arabidopsis*, flavonoid biosynthetic pathway starts with the formation of chalcone by the condensation of three molecules of malonyl-CoA and one molecule of *p*-coumaroyl-CoA, and this step is catalyzed by chalcone synthase (CHS). Chalcone will be subsequently isomerized by chalcone isomerase (CHI) to form flavanone, and flavanone can be catalyzed by flavanone 3-hydroxylase (F3H) to form dihydroflavonol. As the substrate of dihydroflavonol reductase (DFR), dihydroflavonol can be further catalyzed to form leucoanthocyanidins, or to form anthocyanidins by the catalyzation of anthocyanidin synthase (ANS). Subsequently, anthocyanidin will be reduced by anthocyanidin reductase (ANR) to form proanthocyanidin precursors [7] (Fig 1). In *Arabidopsis*, dihydrokaempferol and kaempferol can be hydroxylazed by F3’H to form dihydroquercetin and quercetin on the 3’ position of B-ring. The existence of *F3’5’H* in many other plants, such as tea plant, grape and *Medicago truncatula* could promotes further hydroxylation at the 5’ position in the B-ring of dihydroquercetin and quercetin, to form dihydromyricetin and myricetin [8] (Fig 1). However, no *F3’5’H* gene exists in *Arabidopsis*, which could not synthesize any flavonoid derivatives with trihydroxyl groups on the B-ring as tea plant or grapes that are rich in proanthocyanidins in leave tissues.

The biosynthesis of anthocyanins and proanthocyanidins, and the conversion between them have been hotspots in the study of flavonoid biosynthesis. *Arabidopsis pap1-D* (*production of anthocyanin pigment 1-Dominant*) mutant accumulates massive anthocyanins, in which the MYB type transcription factor PAP1 is activated by using the activation tagging with the enhancer from 35S promoter [9]. A number of genes in anthocyanin biosynthesis, such as *CHS*, *CHI*, *F3H*, *F3’H*, *DFR* and *ANS*, are strongly induced by *PAP1* in *pap1-D* line [10]. Theoretically, more anthocyanin aglycones could provide sufficient substrates for proanthocyanidin production in pathway. In a previous study, ectopic expression of *PAP1* led to massive anthocyanin accumulation in tobacco, and a large amount of proanthocyanidins are accumulated in both mature and young leaves of the *PAP1* and *MtANR* expression plants. Similar results were demonstrated in *Medicago truncatula*, more proanthocyanidins are accumulated in the leaves of *PAP1* and *MtANR* over-expression plants compared with the wild type [11]. These results suggested that ectopic accumulation of proanthocyanidins in plant, in particular in leaves, may be feasible by co-expression of *PAP1* and *ANR* genes.

*ANR* is a key gene for the formation of proanthocyanidin monomers from anthocyanidins, the expression level of *ANR* directly correlates with the accumulation of proanthocyanidins in many plants. The expression pattern of *VvANR* from grapevine (*Vitis vinifera* L. cv *Shiraz*) was positively correlated with the level of proanthocyanidins in grape fruit; the expression level of *VvANR* determined the accumulation of proanthocyanidins in berry development [12]. In blackberry (*Rubus* spp.) fruit, anthocyanins level was gradually increased and proanthocyanidins level was gradually decreased during maturation, the transcript levels of *RuANS*, and *RuANR* were coordinated with the changing patterns of anthocyanins and proanthocyanidins, in which *RuANS* was gradually up-regulated and *RuANR* were down-regulated during the ripening of blackberry fruit [13].

In *Arabidopsis*, seed coat is the main storage part of proanthocyanidins [14], *Arabidopsis* accumulates only epicatechins (3’,4’-hydroxylated) but not galloepicatechins (3’,4’,5’ -hydroxylated) or catechins due to the lack of flavonoid 3′,5′-hydroxylase (F3’5’H) and leucoanthocyanidin reductase (LAR) [15–17]. Different from *Arabidopsis*, tea plant (*Camellia sinensis*) accumulates a great amount of proanthocyanidins in leaves and 15%~25% of them are trihydroxylated proanthocyanidins (3’,4’,5’-hydroxylated) [18]. Heterologous expression of *CsF3’5’H* in yeast revealed that flavanone and flavonol can be hydroxylated by CsF3’5’H, the over-expression of *CsF3’5’H* in tobacco led to the production of delphinidin derivatives in transgenic plants [19]. The presence of *F3’5’H* might be the key for the high accumulation of proanthocyanidins in plants like tea plants and grapes. Meanwhile, *ANRs* genes from tea plant have been characterized to be functional by us in a previous study [20], therefore, it is feasible to re-construct the pathway with genes from the proanthocyanidin-rich tea plant, in another model plant *Arabidopsis* as prove of concept.

In this study, we demonstrated that by the combined over-expression of *CsF3’5’H*, *CsANR2* and *PAP1* in *Arabidopsis*, the accumulation of proanthocyanidins could be changed not only in mature seeds but also in leaves.

## Materials and methods

### Plant material and growth condition

After vernalized under 4°C for 2 days, the wild type *Arabidopsis* (Col-0) and all transgenic lines were germinated and grown in mix soil (vermiculite: nutrient soil = 1:1). The plants were grown under long-day condition (16 h light and 8 h dark) in growth chamber with constant temperature of 23°C.

For the observation of anthocyanins, to avoid obstruction on chlorophyll, *Arabidopsis* were planted on 1/2 MS medium supplied with 100 *μ*M Norflurazon and grown for 4 days after vernalization (4°C for 2 days).

### Cloning and vector constructs

The amplification of the ORF fragment of *CsANR2* (NCBI cDNA accession number: GU992400) and the vector construction of pB2GW7-*CsANR2* were described in our previous study [20]. The ORF fragment of *CsF3’5’H* (NCBI cDNA accession number: DQ194358) was amplified from cDNA by reverse transcription from a tea cDNA library [20], using primers: CsF3’5’H-CF: 5’-CACCATGGCCCTAGACACCGTCTTCCTGC-3’ (the start codon was boxed), CsF3’5’H-R: 5’-TTAAGCAGCATAAGCATTTGGAGGC-3’ (the stop codon was boxed), PCR products were purified and cloned into the Gateway Entry vector pENTR/D-TOPO and confirmed by sequencing. The Entry vector was then transferred into Gateway plant transformation vector pK2GW7 to generate pK2GW7- *CsF3’5’H* by LR reaction.

### *Agrobacterium*-mediated transformation and the crossing of *Arabidopsis*

The pK2GW7-CsF3’5’H, and pB2GW7-CsANR2 were individually transformed into *Agrobacterium tumefaciens* strain GV3101, a single colony for *Agrobacterium*-mediated transformation of *Arabidopsis* was confirmed by PCR. After inoculated into LB liquid medium containing 50 mg/L spectinomycin and 50 mg/L rifampicin, cell suspension was cultured until OD_600_ = 0.6. The cells were collected by centrifugation at 4000 g for 10 min, then resuspended in MS liquid medium for plant transformation. The transformation was performed by using floral-dip method [21].

Before cross-fertilizing, petals and anthers of well-grown female parent plant were gently removed before blooming, then pollenated by the anthers from male parent plant. The pollinated flower was covered by a transparent plastic bag to prevent pollution from other anthers until the silique is formed. For the crossing of *CsF3’5’H* × *pap1-D* and *CsANR2 × pap1-D* over-expression lines, *pap1-D* was used as male parent, the *CsF3’5’H* and *CsANR2* over-expression lines were used as female parent, for the crossing of *CsF3’5’H × CsANR2 × pap1-D* over-expression lines, *CsANR2* over-expression lines were used as male parent, and the *CsF3’5’H* × *pap1-D* over-expression lines were used as female parent.

### Screening and identification of transgenic events

*CsANR2* over-expression lines were screened on solid MS medium (MS salts, 2% (w/v) sucrose, 0.7% (w/v) agar) containing phosphinothricin (PPT, 5 mg/L), *CsF3’5’H* over-expression lines were screened on solid MS medium containing kanamycin (10 mg/L), *CsF3’5’H* × *pap1-D*, *CsANR2* × *pap1-D*, and *CsF3’5’H* × *CsANR2* × *pap1-D* over-expression lines were screened on MS medium containing both PPT (2.5 mg/L) and kanamycin (10 mg/L). Total RNAs of wild type and the transgenic lines were extracted and reverse transcripted to cDNA, and the determination of over-expression lines were confirmed by using RT-PCR with cDNAs and the following primer pairs: CsANR2-F: 5’-ATGGAAGCCCAACCGACAGCTC-3’, CsANR2-R: 5’-TCAATTC-TTCAAAATCCCCTTAGCC-3’ for *CsANR2*, CsF3’5’H-F: 5’-ATGGCCCTAGACACCGTCTTCCTGC-3’, CsF3’5’H-R: 5’-TTAAGCAGCATAAGCATTTGGAGGC-3’ for *CsF3’5’H*, PAP1-F: 5’-ACTTATACCTTTTACAATTTGTTTA-3’, PAP1-R: 5’-CAAACCTATACACAAACGCA-3’ for *PAP1*, PP2A-F: 5’-TATCGGATGACGATTCTTCGTGCAG-3’, PP2A-R: 5’-GCTTGGTCGACTATCGGAATGAGAG-3’ for *PP2A* (*Protein Phosphatase 2A subunit A3*, AT1G13320). *PP2A* gene from *Arabidopsis* was used as reference gene. PCRs were performed by using 2 × *Taq* mastermix (CWBio) and the conditions were 94°C for 2 min, 30 cycles of 94°C for 30 s, 58°C for 30 s, 72°C for 45 s, followed by 72°C of 5 min for final extension.

### Quantitative real-time PCR analysis

Total RNAs were extracted from 21-day-old leaves of *Arabidopsis* lines and the reverse transcription of RNAs was performed by using Transcript One-step gDNA Removal and cDNA Synthesis SuperMix (Transgen Biotech), all cDNAs were diluted to 100 ng/*μ*L. The quantitative real-time PCR was performed by using SYBR Green PCR Master Mix (Takara). The PCR cycling parameters used were as follows: 95°C for 30 s and 40 cycles of 95°C for 5 s, 30 s at 60°C and 30 s at 72°C, followed by a melting curve analysis from 55°C to 95°C. The primer pairs used were as followed: qCsF3’5’H-F: 5’-GTACGATTTGAGTTGTCGCGGT-3’ and qCsF3’5’H-R: 5’-GCCGAAGCCTTGTGTTCCTCTA-3’ for *CsF3’5’H*, qCsANR2-F: 5’-GAGACCCAGGCAATCAGAAAAA-3’ and qCsANR2-R: 5’-ATGACACGTTTAACCGTTCCTG-3’ for *CsANR2*, qAtCHS-F: 5’-CGCATCACCAACAGTGAACAC-3’ and qAtCHS-R: 5’-TCCTCCGTCAGATGCATGTG-3’ for *AtCHS*, qAtDFR-F: 5’-AAACGTTAGCGGAGAAAGCA-3’ and qAtDFR-R: 5’-CCTCGTTCCGAGTGATAGGA-3’ for *AtDFR*, qAtANS-F: 5’-CATCGTGGGTTGGTGAATAA-3’ and qAtANS-R: 5’-GTCCGTGGAGGAAACTTAGC-3’ for *AtANS*, qAtFLS-F: 5’-ACCGTCATGCGTCAATTACA-3’ and qAtFLS-R: 5’-TCAACGCATCACGCTTTAAC-3’ for *AtFLS*, qAtANR-F: 5’-GTGACCGGTCTCAAGGAAAT-3’ and qAtANR-R: 5’-ACAGCAAATGTAGCGACCAG-3’ for *AtANR*, PP2A-F and PP2A-R for *PP2A*. *PP2A* gene from *Arabidopsis* was used as reference gene. Data were collected from three biological replicates. The relative expression level was calculated by using the 2^-△△CT^ method. △C_T_ = C_T target gene_—C_T PP2A_, -△△C_T_ = —(△C_T target gene_—△C_T control_) [22].

### Metabolite analysis

To analyze total flavonoids and flavonols, aerial part of eh 21-day-old seedlings and mature seeds were collected and then grounded in liquid nitrogen. After lyophilized at -50°C for 18 h, 1 ml 80% methanol were firstly added to the samples (200 mg for seedlings and 50 mg for seeds) and sonicated for 30 min, then centrifuged at 10000 g for 30 min after placed at 4°C for 12 h. The absorption of the supernatant was measured at 254 nm. Absorbance values were converted into flavonoids equivalents by using the standard curve of quercetin. The flavonoid extracts were also run on an HPLC 1260 (Agilent) system with an Eclipse XDB-C18 reverse phase column (4.6 × 150 mm, particle size 5 μm) with 150 *μ*L of the supernatant, a linear eluting gradient were consisted of solvent A (0.1% formic acid in water) and solvent B (acetonitrile): 0 min, 10% B; 10 min, 20% B; 15 min, 20% B; 25 min, 50% B; and 30 min, 60% B. A flow rate of 0.8 ml/min were used for the separation of the compounds, the UV-visible absorption from 190–600nm was detected by using a DAD detector (Agilent). Peak areas were converted and calculated into flavonols equivalents by using the standard curve of kaempferol.

For UPLC-MS/MS analysis, I-Class UPLC/Xevo TQ MS (Waters) system was used for chromatographic separation of the samples by using ACQUITY UPLC HSS C_18_ column (1.7 *μ*m, 100×2.1 mm, waters). Solvent A and solvent B was same as mentioned above. Eluting gradient was as followed: 0 min, 95% A; 6 min, 55% A; 7 min, 10% A, 7.1 min– 10 min, 95% A for column balance. Chromatograms were acquired at 254 nm. The flavonoids were employed in positive ion (PI) and in negative ion (NI) mode, MS detection conditions were as followed: desolvation temperature: 400°C, desolvation gas (N_2_) flow: 800 L/h, cone gas flow: 50 L/h, cone voltage: 30 V and capillary voltage, 3 kV for PI mode, cone voltage -60 V and capillary voltage 2 kV for NI mode, scan range: 100–1000 (m/z).

For the analysis of anthocyanins, leaves of 30-day-old plants were collected and then lyophilized, then the samples (200 mg) were extracted with methanol (contains 0.1% hydrochloric acid, by vol.) and sonicated for 30 min. After centrifuged at 10000 g for 30 min, the supernatant was extracted and chloroform/water were added to remove chlorophyll. The absorption of the products was measured at 550 nm. Data were collected from three biological replicates. The anthocyanins level was calculated by using the formula: C_anthocyanins_ = OD_550_/ε × V/m × 10^6^; in which ε = 4.62 × 10^6^, V = 4mL, m = 200 mg.

To analyze the soluble proanthocyanidins, leaves of 30-day-old plants and mature seeds were collected and grounded in liquid nitrogen and lyophilized in -50°C for 18 h. DMACA (*p*-dimethylaminocinnamaldehyde) is an aldehyde dye, and proanthocyanidins can react with DMACA to form a blue stain. For the DMACA detection method, 500 *μ*L 70% acetone (contain 0.5% acetic acid by vol.) were firstly added to the samples (200 mg for leaves and 50 mg for mature seeds) and sonicated for 30 min, and then centrifuged at 3000 g for 10 min, the extraction was repeated twice and the supernatants were all collected. After extracted by chloroform and n-hexane to remove the chlorophyll, 760 *μ*L of 0.2% (w/v) DMACA solution were added into 40 *μ*L of the supernatants and the absorption were measured at 640 nm within 15 min; for the butanol-HCl reaction, 760 *μ*L of the butanol-HCl (95%-5%, by vol.) reagent was added into 40 *μ*L of the supernatants and the absorption 1 (A1) of the samples were measured at 550 nm, then the samples were boiled at 95°C for 1 h and measured at 550nm again as absorption 2 (A2), the practical absorption of the samples was calculated as A2 minus A1. Absorbance values were converted into proanthocyanidins equivalents by using the standard curve of procyanidin B1. The remained residues of these steps were all collected for insoluble proanthocyanidins measurement.

To analyze the insoluble proanthocyanidins, the residues described above were dried using a vacuum drier, then 1 mL of the butanol-HCl (95%-5%, by vol.) reagent was added and sonicated for 60 min. After centrifuged at 3000 g for 10 min, the absorption (A1) of the samples were measured at 550 nm. Then the samples were boiled at 95°C for 1 h and measured at 550 nm again as absorption 2 (A2). The practical absorption of the samples was calculated as A2 minus A1. Absorbance values were converted into proanthocyanidins equivalents by using the standard curve of procyanidin B1.

## Results

### Analyses of anthocyanins in transgenic *Arabidopsis*

**Fig 2:**
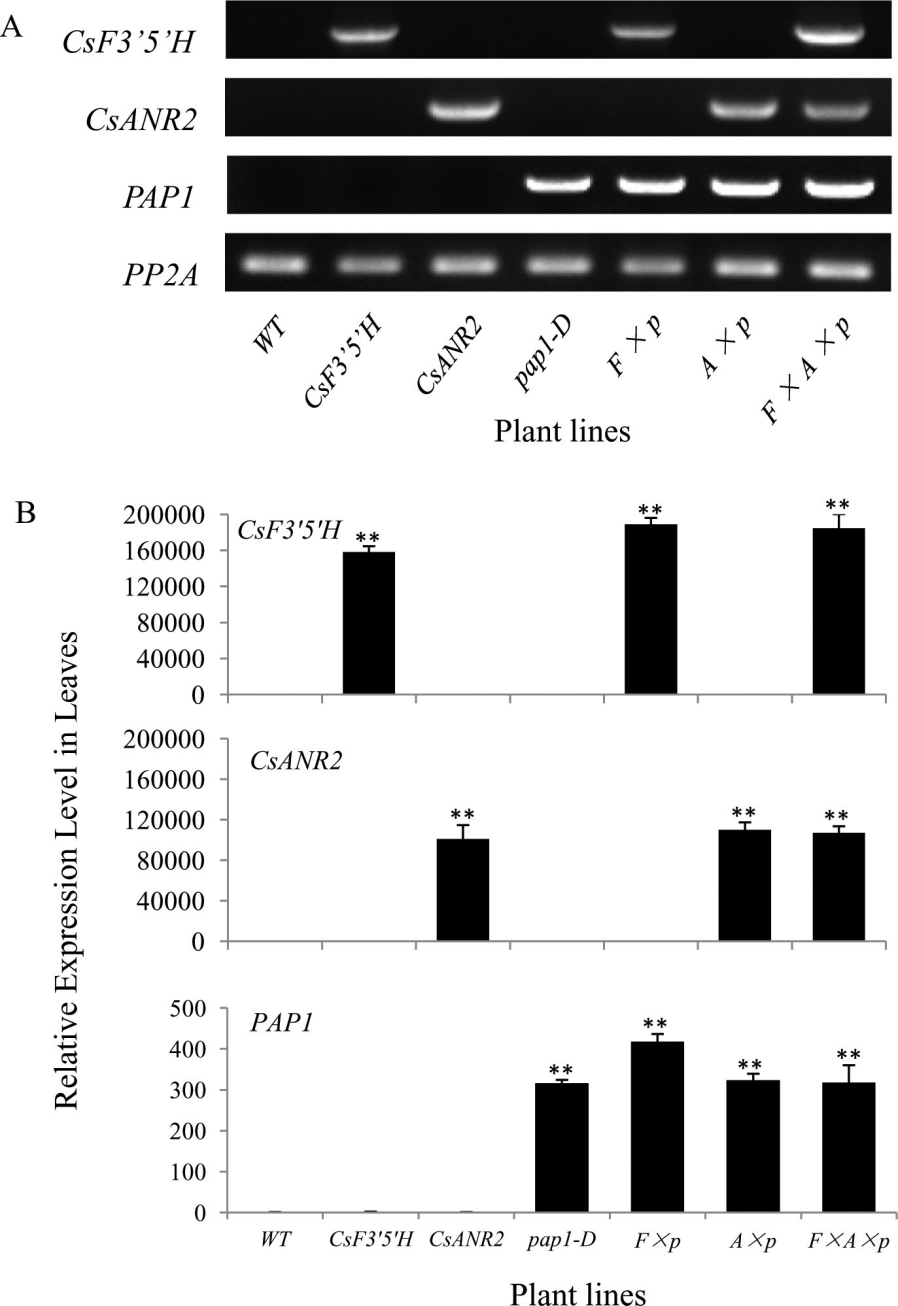
Detection of the expression levels of genes in flavonoid pathway in *Arabidopsis* leaves. (A) Molecular characterization of transgenic lines detected by RT-PCR; (B) Relative transcript levels of *CsF3’5’H*, *CsANR2* and *PAP1* in *CsF3’5’H*, *CsANR2*, *pap1-D*, *CsF3’5’H × pap1-D* (*F × p*), *CsANR2 × pap1-D (A × p*) and *CsF3’5’H × CsANR2 × pap1-D* (*F × A × p*) over-expression lines by qRT-PCR analysis. Data are presented as mean ± SD, and significant differences were calculated by one-way ANOVA (n = 3, *P<0.05, **P<0.01).

To verify the functions of *CsF3’5’H* and *CsANR2 in vivo*, we individually transformed *CsF3’5’H* and *CsANR2* into the wild type *Arabidopsis* (Columbia-0), 42 *CsF3’5’H* over-expression lines and 45 *CsANR2* over-expression lines were obtained, respectively. The over-expression lines of these two genes were verified by both RT-PCR and qRT-PCR, one line with the highest expression level of 42 *CsF3’5’H* over-expression lines or 45 *CsANR2* over-expression lines were identified, respectively, and further used for the following cross-fertilization assays. We pollinated the stigma of the *CsF3’5’H* over-expression line or the *CsANR2* over-expression line with *pap1-D* line to obtain *CsF3’5’H × pap1-D* and *CsANR2× pap1-D* double over-expression lines, respectively. At last, the stigma of one *CsF3’5’H × pap1-D* over-expression line was pollinated with pollens from one *CsANR2* over-expression line to obtain the *CsF3’5’H × CsANR2 × pap1-D* triple over-expression lines. The expression levels of the three genes *CsF3’5’H*, *CsANR2* and *PAP1* in all the transgenic plants were further confirmed by RT-PCR and qRT-PCR (Fig 2).

**Fig 3:**
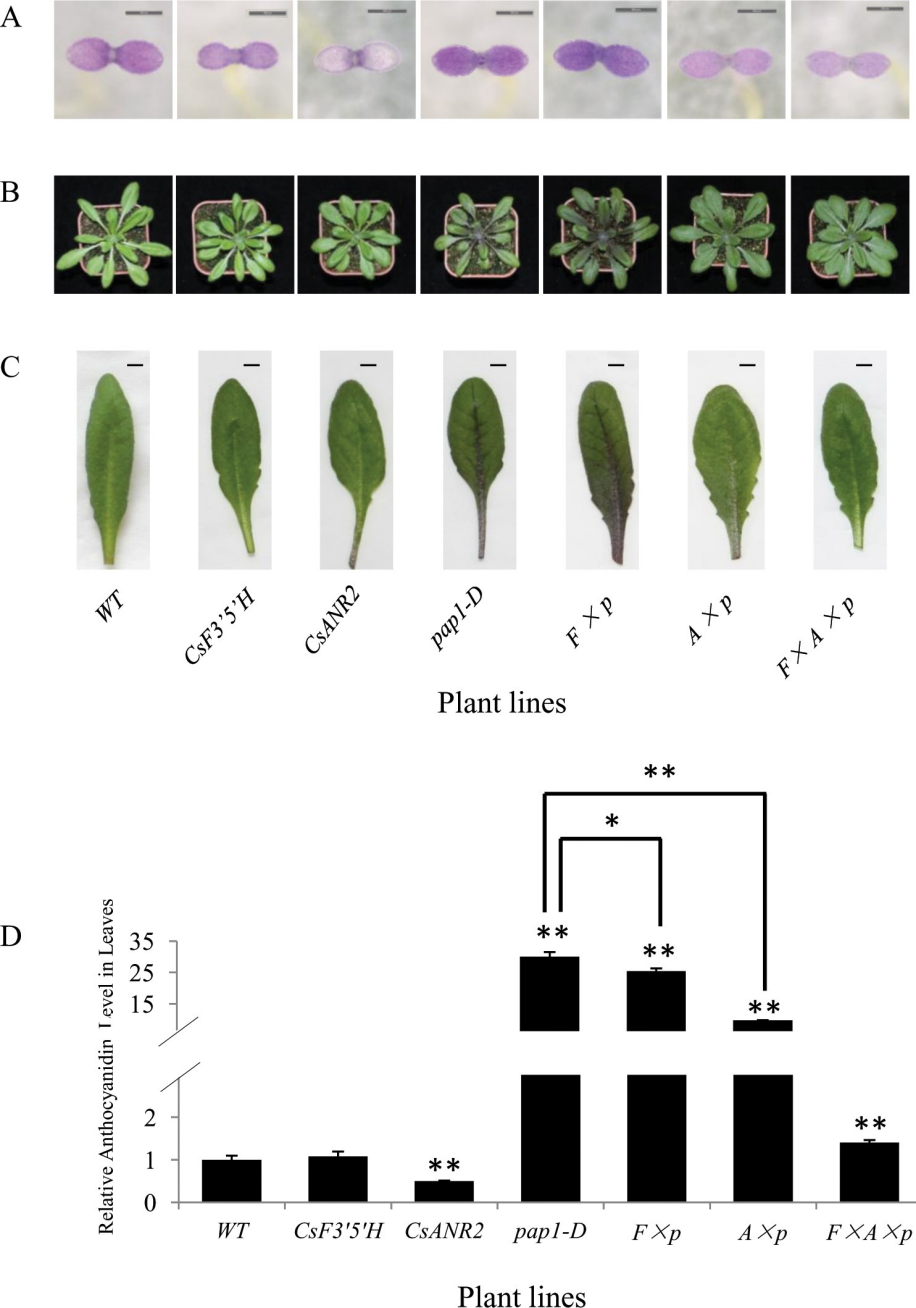
The phenotype and anthocyanins level of the transgenic and wild type *Arabidopsis*. (A) Anthocyanin accumulation in 4-day-old seedling (Bars = 500 *μ*m). (B) Anthocyanin accumulation in 30-day-old aerial parts. (C) Anthocyanin accumulation in 30-day-old leaves (Bars = 5mm). (D) Relative level of anthocyanins in 30-day-old aerial parts, anthocyanin level of wild type control (Col-0, 0.97 mg/g dry weight) was set as a value of 1.0. Data are presented as mean ±SD, and significant differences were calculated by one-way ANOVA (n = 3, *P<0.05, **P<0.01).

First, the accumulation of anthocyanins was analyzed in the 4-day-old seedlings, we found that the *CsF3’5’H* over-expression lines showed light purple cotyledons as the wild type, but the cotyledons of the *CsANR2* over-expression lines were pale and slightly purple on the edge. The *CsF3’5’H × pap1-D* over-expression lines showed the same deep purple as *pap1-D*. Leave color of the *CsANR2 × pap1-D* over-expression lines and the *CsF3’5’H × CsANR2 × pap1-D* over-expression lines was lighter than that of the wild type (Fig 3A).

In the leaves of 30-day-old seedlings of the *CsF3’5’H* and *CsF3’5’H × CsANR2 × pap1-D* over-expression lines, the petioles were light purple and the blades were green, which was nearly the same color as the wild type. The petioles and the blades were all green in the leaves of *CsANR2* over-expression lines. Leaves in *CsF3’5’H × pap1-D* over-expression lines and *pap1-D* were intensively purple than the wild type. The leaves of *CsANR2 × pap1-D* over-expression lines were slightly purple (both petioles and blades) than the wild type, but not as deep as the *CsF3’5’H × pap1-D* over-expression lines or *pap1-D* lines (Fig 3B and 3C), indicating that anthocyanins might be converted to proanthocyanidin by CsANR2.

To further quantitatively determine anthocyanins content, anthocyanins in wild type and the other six representative transgenic lines were extracted and measured. As shown in Fig 3D, anthocyanins level in *CsANR2* over-expression lines was only half of the wild type, and anthocyanins level in *CsF3’5’H* over-expression lines was nearly the same as the wild type. However, anthocyanins level in *pap1-D* was 30-fold higher than the wild type, anthocyanins level in*CsF3’5’H × pap1-D* line was decreased by 25-fold compared to the wild type. Anthocyanins level in *CsANR2 × pap1-D* over-expression lines decreased more obviously. When *CsF3’5’H* and *CsANR2* were co-expressed in *pap1-D*, anthocyanins level was dramatically decreased to only 1.4-fold of the wild type (Fig 3D).

Taken together, anthocyanins level in *CsF3’5’H* over-expression lines showed no difference from the wild type; the anthocyanins level of *CsF3’5’H × pap1-D* over-expression lines was obviously reduced compared to the *pap1-D* line, and the over-expression of *CsANR2* could significantly reduce the level of anthocyanins in *pap1-D*. More importantly, anthocyanins level could be largely reduced in *CsF3’5’H × CsANR2 × pap1-D* over-expression lines, indicating that co-expression of *CsF3’5’H* and *CsANR2* could significantly reduce anthocyanin levels in the presence of PAP1.

### Analyses of proanthocyanidins in leaves of transgenic *Arabidopsis*

Proanthocyanidins are accumulated mainly in *Arabidopsis* seeds [23], and the co-expression of *MtANR* and *PAP1* lead to a huge increase of proanthocyanidins in tobacco leaves [11]. To exam whether co-expression of *CsF3’5’H*, *CsANR2* and *PAP1* could affect proanthocyanidins biosynthesis in leaves, leaves of transgenic *Arabidopsis* were collected for proanthocyanidins measurement.

**Fig 4:**
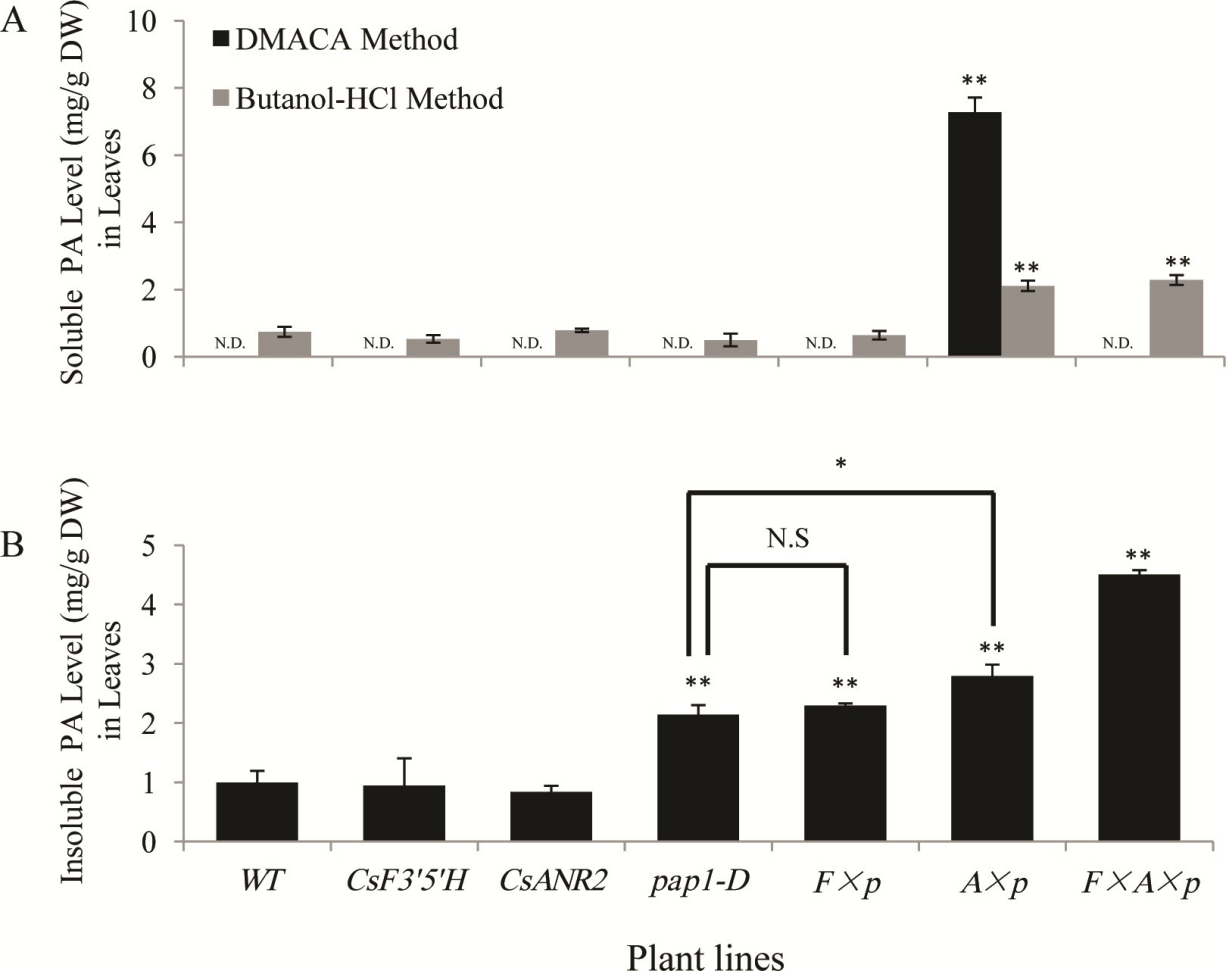
The level of proanthocyanidins in 30-day-old leaves of transgenic and wild type *Arabidopsis*. (A) Level of soluble proanthocyanidins in 30-day-old leaves (N.D. Not detected). (B) Level of insoluble proanthocyanidins in 30-day-old leaves. Data are presented as mean ±SD, and significant differences were calculated by one-way ANOVA (n = 3, *P<0.05, **P<0.01).

The DMACA-reactive soluble proanthocyanidins were only found in *CsANR2 × pap1-D* over-expression lines and no detectable soluble proanthocyanidins were detected in the other six lines by using DMACA method. When detected by using butanol-HCl method, the content of soluble proanthocyanidins in *CsANR2 × pap1-D* and *CsF3’5’H × CsANR2 × pap1-D* over-expression lines was approximately 3-fold (2.11 mg/g DW and 2.28 mg/g DW, respectively) higher than in the wild type (0.74 mg/g DW) and the level of butanol-HCl reactive proanthocyanidins in other 4 lines stayed unchanged (Fig 4A).

The insoluble proanthocyanidins level in the *CsF3’5’H* over-expression lines, *CsANR2* over-expression lines showed no obvious difference (1.91 mg/g DW and 1.70 mg/g DW, respectively) with the wild type (2.02 mg/g DW). Over-expression of *CsANR2* in *pap1-D* led to a 20% increase in insoluble proanthocyanidins compared to the *pap1-D* line (5.65 mg/g DW and 4.64 mg/g DW, respectively), and obvious increase was found in the *CsF3’5’H × pap1-D* over-expression lines (4.33 mg/g DW). Insoluble proanthocyanidins level in the *CsF3’5’H× CsANR2 × pap1-D* co-expression line was significantly up-regulated to 9.11 mg/g (DW), which was 4-fold higher than the wild type and 2-fold than the *pap1-D* line (Fig 4B).

In conclusion, proanthocyanidins were significantly increased in leaves of *pap1-D* when *CsANR2* was over-expressed, and the over-expression of *CsF3’5’H* alone or *pap1-D* did not increase proanthocyanidin accumulation, but co-expression of *CsF3’5’H* and *CsANR2* significantly increased proanthocyanidins level in leaves of the *pap1-D* line.

### Analysis of proanthocyanidins in mature seeds of the transgenic *Arabidopsis*

Owing to the increase of proanthocyanidins in 30-day-old leaves of the transgenic *Arabidopsis*, we further detected the accumulation of proanthocyanidins in mature seeds.

**Fig 5:**
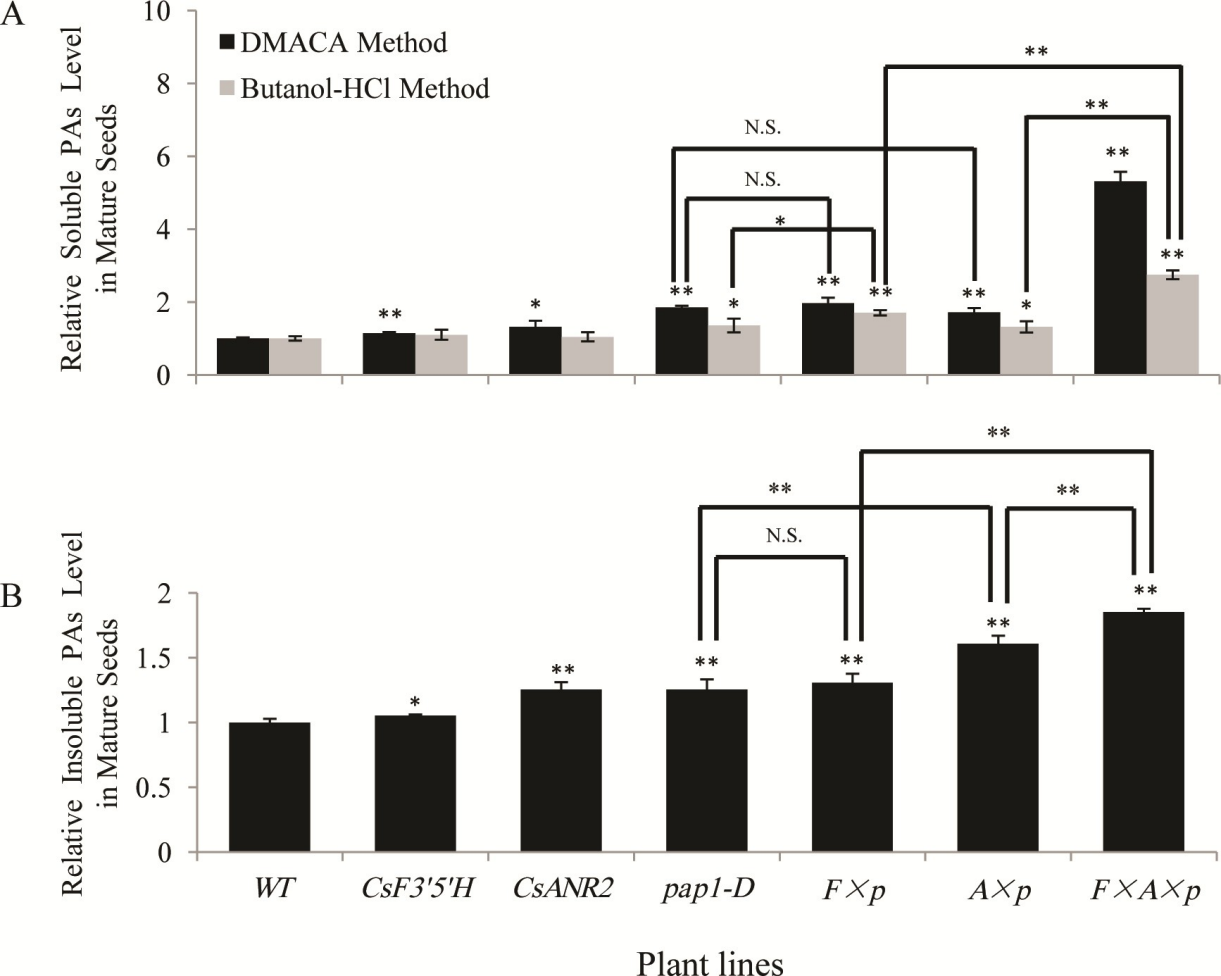
Proanthocyanidins level in mature seeds of the transgenic and wild type *Arabidopsis*. (A) Soluble proanthocyanidins level in mature seeds of the wild type detected with DMACA method (0.59 mg/g dry weight) and butanol-HCl method (1.85 mg/g dry weight) were set as a value of 1.0, respectively. (B) Insoluble proanthocyanidins level in mature seeds of the wild type control (68.15 mg/g dry weight) was set as a value of 1.0. Data are presented as mean ±SD, and significant differences were calculated by one-way ANOVA (n = 3, *P<0.05, **P<0.01).

The level of soluble proanthocyanidins in *CsF3’5’H × CsANR2* over-expression lines was slightly increased (1.14-fold and 1.32-fold with DMACA method, 1.10-fold and 1.03-fold with butanol-HCl method, respectively) compared to the wild type. The level of proanthocyanidins were slightly increased by the over-expression of *CsF3’5’H* using butanol-HCl method (1.70-fold) in *pap1-D*, while other lines stayed the same level compared to *pap1-D* that was 1.36-fold of the wild type. In addition, the level of soluble proanthocyanidins was substantially increased in *CsF3’5’H × CsANR2 × pap1-D* over-expression lines, the DMACA-reactive proanthocyanidins were 4.31-fold higher than the wild type, and the butanol-HCl-reactive proanthocyanidins was 2.75-fold higher than the wild type (Fig 5A).

We further detected the level of insoluble proanthocyanidins and found that the accumulation of insoluble proanthocyanidins was slightly increased in *CsF3’5’H* over-expression lines and *CsANR2* over-expression lines (1.06-fold and 1.26-fold, respectively) than in the wild type. The level of insoluble proanthocyanidins in *CsF3’5’H × pap1-D* over-expression lines did not show obvious change as in *pap1-D*, and the *CsANR2 × pap1-D* over-expression lines had nearly 30% increase in insoluble proanthocyanidins. The accumulation of insoluble proanthocyanidins was significantly increased in *CsF3’5’H × CsANR2 × pap1-D* over-expression lines, which was approximate 85% increase compared to the wild type and nearly 50% increase compared to the *pap1-D* line (Fig 5B).

In summary, in mature seeds, the over-expression of *CsF3’5’H* or *CsANR2* has no strong effect on the level of soluble proanthocyanidins, the level of insoluble proanthocyanidins could be up-regulated by *CsANR2*. The co-expression of *CsF3’5’H* and *CsANR2* in *pap1-D* led to a huge increase in both soluble and insoluble proanthocyanidins levels.

### Analysis of total flavonoids and flavonols in transgenic *Arabidopsis*

**Fig 6:**
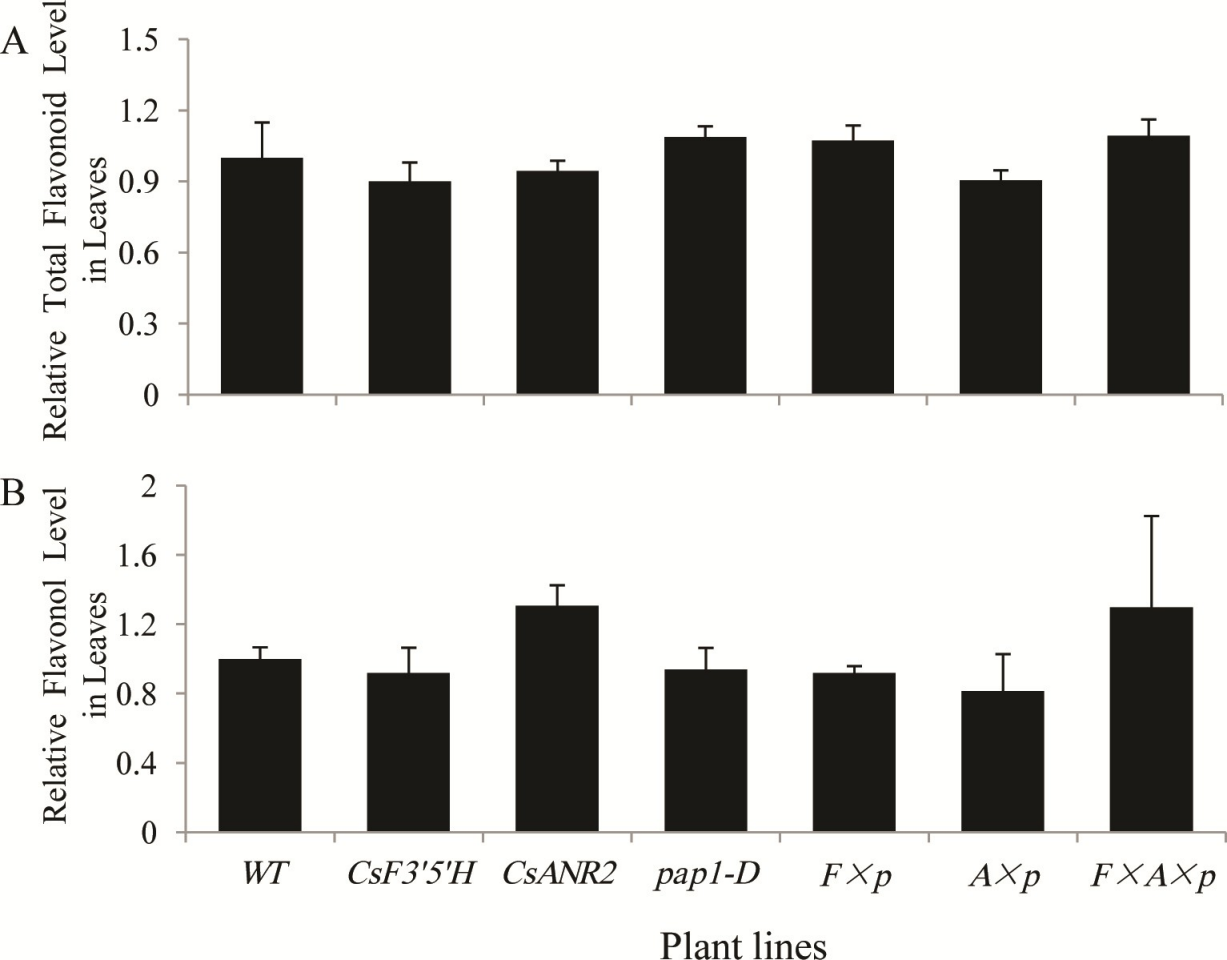
Total flavonoids and total flavanols levels in *Arabidopsis* leaves. (A) Relative total flavonoid level in 21-day-old aerial part. Flavonoid level in wild type control (31.06 mg/g dry weight) were set as a value of 1.0. (B) Relative flavonol level in 21-day-old aerial part. Flavonol level in wild type control (191.17 *μ*g/g dry weight) were set as a value of 1.0. Data are presented as mean ±SD, and significant differences were calculated by one-way ANOVA (n = 3, *P<0.05, **P<0.01).

To exam whether total flavonoids of the transgenic lines were affected, leaves of all the transgenic lines and the wild type were collected for analysis. The level of total flavonoids in all six transgenic lines did not show significant changes compared to the wild type (Fig 6A).

**Fig 7:**
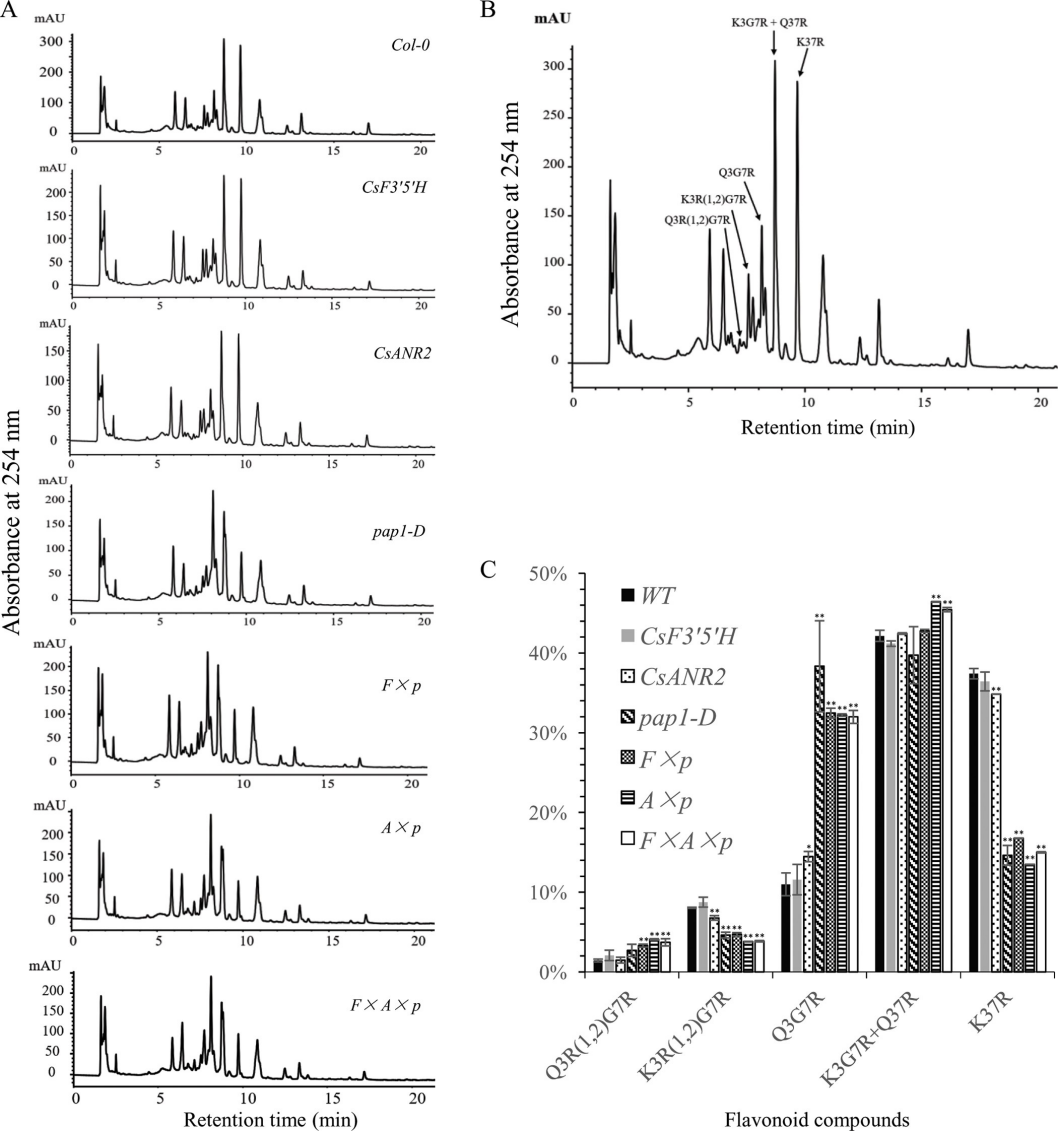
Determination and quantification of flavonoid profiles by using HPLC chromatograms in transgenic and wild type *Arabidopsis*. (A) HPLC chromatograms of flavonoid profiles in the transgenic lines and wild type. (B) Characterization of flavanols in wild type *Arabidopsis*, Abbreviations: Q3R(1,2)G7R: Quercetin-3-*O*-α-l-rhamnopyranosyl(1,2)-β-d-glucopyranoside-7-*O*-α-l-rhamnopyranoside; K3R(1,2)G7R: Kaempferol-3-*O*-α-l-rhamnopyranosyl(1,2)-β-d-glucopyranoside-7-*O*-α-l-rhamnopyranoside; Q3G7R: Quercetin-3-*O*-β-glucopyranosyl-7-*O*-α-rhamnopyranoside; K3G7R: Kaempferol-3-*O*-β-glucopyranosyl-7-*O*-α-rhamnopyranoside; Q37R: Quercetin-3,7-*O*-α-l-di-rhamnopyranoside; K37R: Kaempferol 3,7-*O*-dirhamnopyranoside. (C) Relative level of Q3R(1,2)G7R, K3R(1,2)G7R, Q3G7R, K3G7R+ Q37R and K37R. Data are presented as mean ±SD, and significant differences were calculated by one-way ANOVA (n = 3, *P<0.05, **P<0.01).

Flavonols of all six transgenic lines and the wild type were detected on UPLC-MS(Fig 7). The level of total flavonols did not show any obvious changes in all six transgenic lines (Fig 6B), but the composition of flavonols was obviously changed in all over-expression lines when *PAP1* was over-expressed. As revealed in Fig 7C, the level of quercetin-3-*O*-β-glucopyranosyl-7-*O*-α-rhamnopyranoside (Q3G7R) was increased in *pap1-D*, *CsF3’5’H × pap1-D*, *CsANR2 × pap1-D* and *CsF3’5’H × CsANR2 × pap1-D* over-expression lines, while the level of kaempferol 3,7-*O*-dirhamnopyranoside (K37R) was decreased. Except for the changes in Q3G7R and K37R, no significant changes in other flavonols were found in all six transgenic lines in comparison with the wild type. These results indicated that *PAP1* also affects flavonol profiles besides flavonol content.

### Analyses of transcript level of key genes in the transgenic *Arabidopsis*

*PAP1* has been demonstrated as a key transcription factor in flavonoid pathway and the transcript level of many pathway genes could be up-regulated by *PAP1* [10]. We further investigated whether the expression level of other flavonoid pathway genes was affected by the over-expression of *CsF3’5’H*, *CsANR2* and/or *PAP1*.

**Fig 8:**
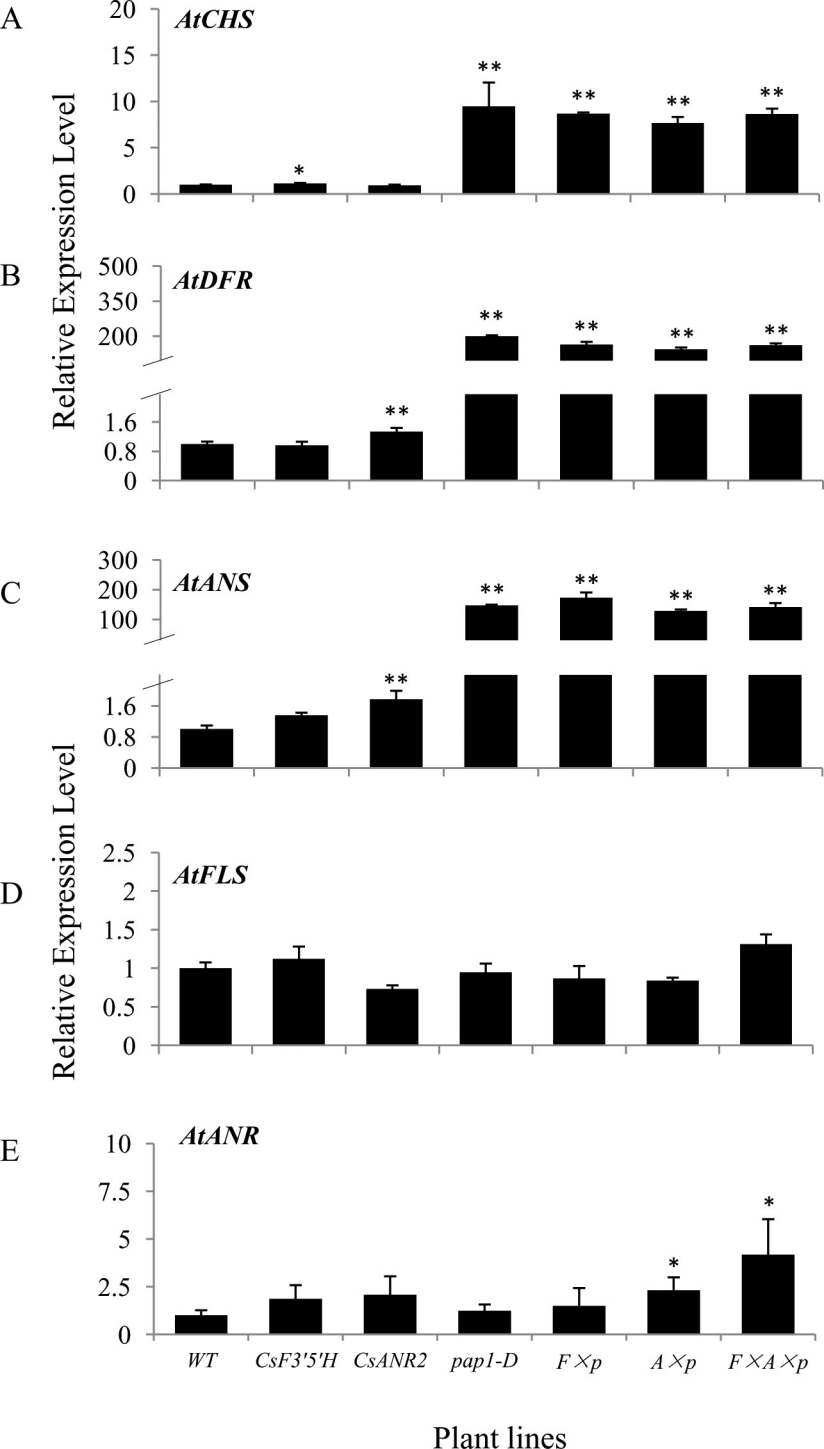
Relative transcript levels of endogenous *CHS*, *DFR*, *ANS*, *FLS* and *ANR* genes in 30-day-old leaves compared with wild type *Arabidopsis* as detected by qRT-PCR. Data are presented as mean ±SD, and significant differences were calculated by one-way ANOVA (n = 3, *P<0.05, **P<0.01).

As the first gene of the flavonoid pathway, *CHS*, was strongly up-regulated when *PAP1* was expressed. The transcript level of *CHS* in *pap1-D* was nearly 10-fold higher than of the wild type, and it was increased by approximately 7-fold in *CsF3’5’H × pap1-D*, *CsANR2 × pap1-D* and *CsF3’5’H × CsANR2 × pap1-D* over-expression lines. The transcript level of *CHS* in the *CsF3’5’H* and *CsANR2* over-expression lines did not show significant difference with the wild type (Fig 8A).

*DFR* and *ANS* are critical genes for anthocyanins biosynthesis. The expression level of *DFR* was increased almost 200-fold in *pap1-D* compared to the wild type, as the transcript level of *DFR* in *CsF3’5’H × pap1-D*, *CsANR2 × pap1-D* and *CsF3’5’H × CsANR2 × pap1-D* over-expression lines was approximately increased 150-fold, which is mainly due to the presence of *PAP1*. The transcripts of *DFR* in *CsF3’5’H* over-expression line or in *CsANR2* over-expression line did not show obvious difference from the wild type. The transcript level of *ANS* were elevated more than 100-fold in all 4 lines due to the over-expression of *PAP1*, especially more than 170-fold in *CsF3’5’H × pap1-D* over-expression line. In *CsF3’5’H* over-expression line and *CsANR2* over-expression line, the transcript level of *ANS* did increase significantly (1.36-fold and 1.78-fold, respectively, Fig 8B and 8C).

The transcript level of *FLS* is directly related to the level of flavonols in *Arabidopsis*. As shown in Fig 8D, the transcript level of *FLS* in all the transgenic lines was almost unchanged compared to the wild type, which was positively correlated to the total flavonols level in all transgenic lines.

*ANR* is one of the most important genes in the biosynthesis of proanthocyanidins. The transcript level of endogenous *ANR* gene in *CsANR2*, *CsANR2 × pap1-D* and *CsF3’5’H × CsANR2 × pap1-D* over-expression lines was slightly increased (2.08-fold, 2.32-fold and 4.18-fold, respectively) compared to the wild type, but the transcripts of endogenous *ANR* in *CsF3’5’H*, *pap1-D*, and *CsF3’5’H ×pap1-D* over-expression lines did not show significant change (Fig 8E).

These data indicated that the transcript level of endogenous *CHS*, *DFR* and *ANS* was strongly up-regulated by *PAP1*, which is correlated with anthocyanin levels (Fig 3D). The transcript level of endogenous *ANR* slightly increased while *CsANR2* existed, but the expression of *FLS* was not affected by *CsF3’5’H*, *CsANR2* or *PAP1*.

## Discussion

### The redirection of metabolic flux to proanthocyanidin pathway

Anthocyanidins could be catalyzed by ANR to form proanthocyanidin monomers; presumably, in the presence of ANR, more proanthocyanidins will be produced when there is massive anthocyanidins accumulated, but the competition between the biosynthesis of proanthocyanidins and anthocyanins could be a block for the engineering of proanthocyanidins in plant as they share the same early biosynthetic pathway (Fig 1) [17,24]. Anthocyanidins could be reduced to form proanthocyanidin monomers, meanwhile, they could also be modified to form anthocyanins as conjugates. The *ANR* mutant *ban* in *Arabidopsis* accumulated more anthocyanins compared to the wild type, and the mutant of anthocyanidin UDP-glucosyltransferase gene *UGT78D2* accumulated more proanthocyanidins [25].

In our study, total flavonoids level did not change in all transgenic lines compared to the wild type (Fig 6A), but the levels of two main final products—anthocyanins and proanthocyanidins obviously changed. Anthocyanins level in *pap1-D* was much higher than in the wild type, but decreased by the introduction of *CsF3’5’H* or *CsANR2* (Fig 3), which were mainly ascribed to the heterologous expression of *CsF3’5’H* and *CsANR2* since the expression of endogenous *AtANS* and *AtANR* were not significantly affected (Fig 8C and 8E). Therefore, by the over-expression of *CsF3’5’H* and *CsANR2*, proanthocyanidins level in leaves and mature seeds were increased at various degree (Figs 4 and 5).

It should also be pointed out that though *CsF3’5’H* and *CsANR2* were both driven by the 35S promoter, proanthocyanidins were affected distinctly in different parts. Proanthocyanidins level was increased in leaves of the *CsANR2 × pap1-D* over-expression lines (Fig 4), levels of butanol-HCl reactive soluble proanthocyanidins in mature seeds of the *CsANR2 × pap1-D* over-expression lines were not changed and those of the CsF3’*5’H × pap1-D* over-expression lines were increased (Fig 5A). These results indicated that, total flavonoids level could not be affected by the over-expression of *CsF3’5’H* or *CsANR2*, but their components were changed by redirecting the metabolic flux from anthocyanins to proanthocyanidins. Therefore, it is possible that the ectopic expression of the specific genes could redirect the metabolic flux in *Arabidopsis*.

### The synergistic effects of CsANR2 and CsF3’5’H in *Arabidopsis*

It is well known that massive proanthocyanidins were accumulated in tea leaves but none in *Arabidopsis* leave [14,18], the different accumulation pattern between these two plants was interesting for investigation. The functions of *CsANR2* and *CsF3’5’H* were previously identified as two structural genes in flavonoids biosynthesis in tea plant [19,26]. It is reported that the presence of CsANRs in enzyme preparations could catalyze cyanidins and delphinidin into epicatechin and epigallocatechin in tea plants [26], the ectopic over-expression of *CsANR2* in tobacco leaves caused the reduction of anthocyanins and the increase of proanthocyanidins. Meanwhile, by the over-expression of *CsANR2*, the level of soluble proanthocyanidins were up-regulated in the hairy roots of *Medicago truncatula* alone, with the decrease of anthocyanins [20]. Heterologous expression of *CsF3’5’H* in yeast showed that naringenin was converted into both 3’4’- and 3’4’5’-forms, the over-expression of *CsF3’5’H* in tobaccos could produce intensively purple flowers by increasing the content of anthocyanins [19].

However, previous work was mainly focused on the functions of these genes alone but not their synergistic effects, therefore, we further investigated the effect of their co-expression. In our study, we found that the synergistic effect of *CsF3’5’H* and *CsANR2* appeared to be very strong by co-expression in *Arabidopsis*, the effects on both the decreasing of anthocyanins and the increasing of proanthocyanidins were more significant compared to the expression of individual *CsF3’5’H* or *CsANR2*, it might be interesting to further investigate the in-depth mechanism of their synergy in future work.

In spite of the obvious effects of *CsF3’5’H* and *CsANR2* on anthocyanins and proanthocyanidins, it is unfortunately that we did not detect trihydroxylated flavonoids even by mass spectrum (Fig 7), which may be due to the transit conversion of trihydroxylated intermediates to other products, or they were all accumulated in form of possible insoluble proanthocyanidin (trihydroxylated monomers) that is hard for detection. Nevertheless, the heterologous expression will still be a big bottleneck for the genes to be fully functional in plant.

### Engineering of proanthocyanidins in plant leaves

The co-expression of *PAP1*, *TT2* and maize TF *Lc* resulted in detectable proanthocyanidins in the leaves of *Arabidopsis*, but the plant could only survive no more than 2 weeks after germination [27]. In tobacco leaves, the level of anthocyanins in *PAP1* and *MtANR* co-expression lines was decreased compared to the *PAP1* expression lines but higher than wild type, and there are detectable proanthocyanidins in both leaves and corollas [11]. In our study, the expression of *CsF3’5’H*, *CsANR2* or *PAP1* alone did not show a significant effects on proanthocyanidin levels, but the co-expression of *PAP1* and *CsANR2* in *Arabidopsis* led to a massive accumulation of DMACA-reactive proanthocyanidins in leaves (Fig 4A), and the plants grew normally and generate progeny. As the *PAP1* and *CsANR2* co-expressing lines accumulated DMACA-reactive proanthocyanidins in leaves, we did not find any accumulation of DMACA-reactive proanthocyanidins in *CsF3’5’H × CsANR2 × pap1-D* co-expressing lines (Fig 4A). It should be pointed out that the content of anthocyanidins in leaves may obstruct the measurements of butanol-HCl reactions and weakly react with DMACA.

The manipulation of proanthocyanidins in *Arabidopsis* by co-expression of *CsF3’5’H*, *PAP1* and *CsANR2* caused changes at different levels and in different tissues, especially in aerial parts of *Arabidopsis*. Although many genes, biosynthetic pathway genes or regulator genes were used to increase proanthocyanidins content in aerial parts of plants, in particular legume crops like alfalfa and white clover, but the results remain still unsatisfactory. Therefore, our study could offer a novel solution for the improvement of proanthocyanidins in a heterologous plant species. Nevertheless, more studies are required to discover more efficient genes for high proanthocyanidins engineering in forage crops.

## Supporting information

S1 Fig Detection of the expression levels of the transgenes in *Arabidopsis* leaves by RT-PCR. The genes used for detection are *CsF3'5'H*, *ANR*, *PAP1* and *PP2A* from left to right. Sample names for each lane are (from left to right): 1. molecular marker; 2. *CsF3’5’H*-over-expression line; 3. *CsANR2*-over-expression line; 4. *pap1-*D mutant line; 5. *CsF3’5’H* × *pap1*-D (F × p) double crossing line; 6. *CsANR2* × *pap1*-D (A × p) double crossing line; 7. *CsF3’5’H* × *CsANR2* × *pap1*-D (F × A × p) triple crossing line. 8. The wild type line as control.
(DOCX)
