# Supplementary material for: Stacking triple genes increased proanthocyanidins level in Arabidopsis thaliana
Source: PLoS One. 2020 Jun 25;15(6):e0234799. doi: 10.1371/journal.pone.0234799 (PMC7316333; doi:10.1371/journal.pone.0234799)
Supplement: S1 Fig — The genes used for detection are CsF3'5'H, ANR, PAP1 and PP2A from left to right. Sample names for each lane are (from left to right): 1. molecular marker; 2. CsF3’5’H-over-expression line; 3. CsANR2-over-expression line; 4. pap1-D mutant line; 5. CsF3’5’H × pap1-D (F × p) double crossing line; 6. CsANR2 × pap1-D (A × p) double crossing line; 7. CsF3’5’H × CsANR2 × pap1-D (F × A × p) triple crossing line. 8. The wild type line as control. (DOCX) [file pone.0234799.s001.docx]

**Supporting Information**

***CsF3'5'H ANR PAP1 PP2A***

**
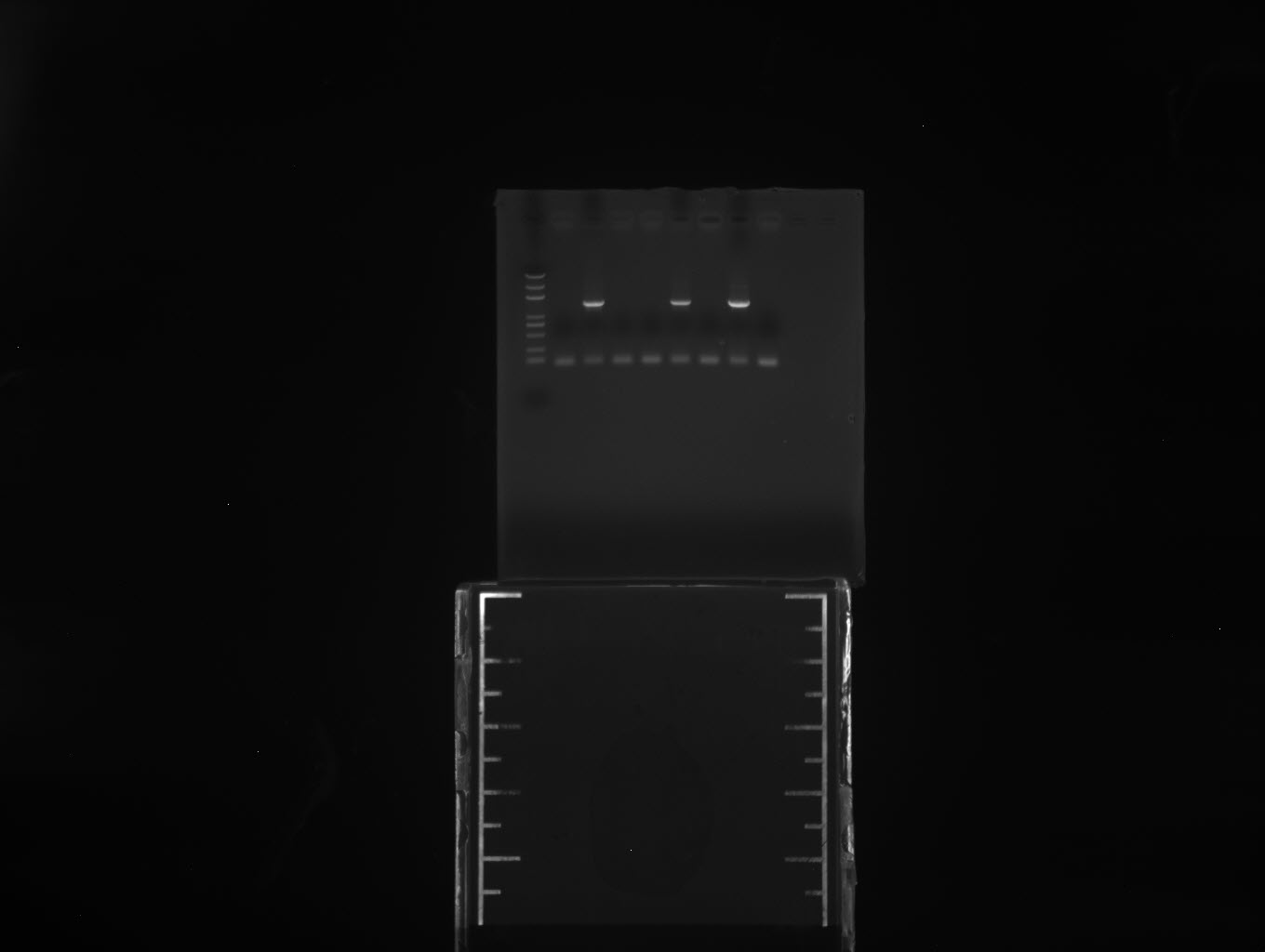

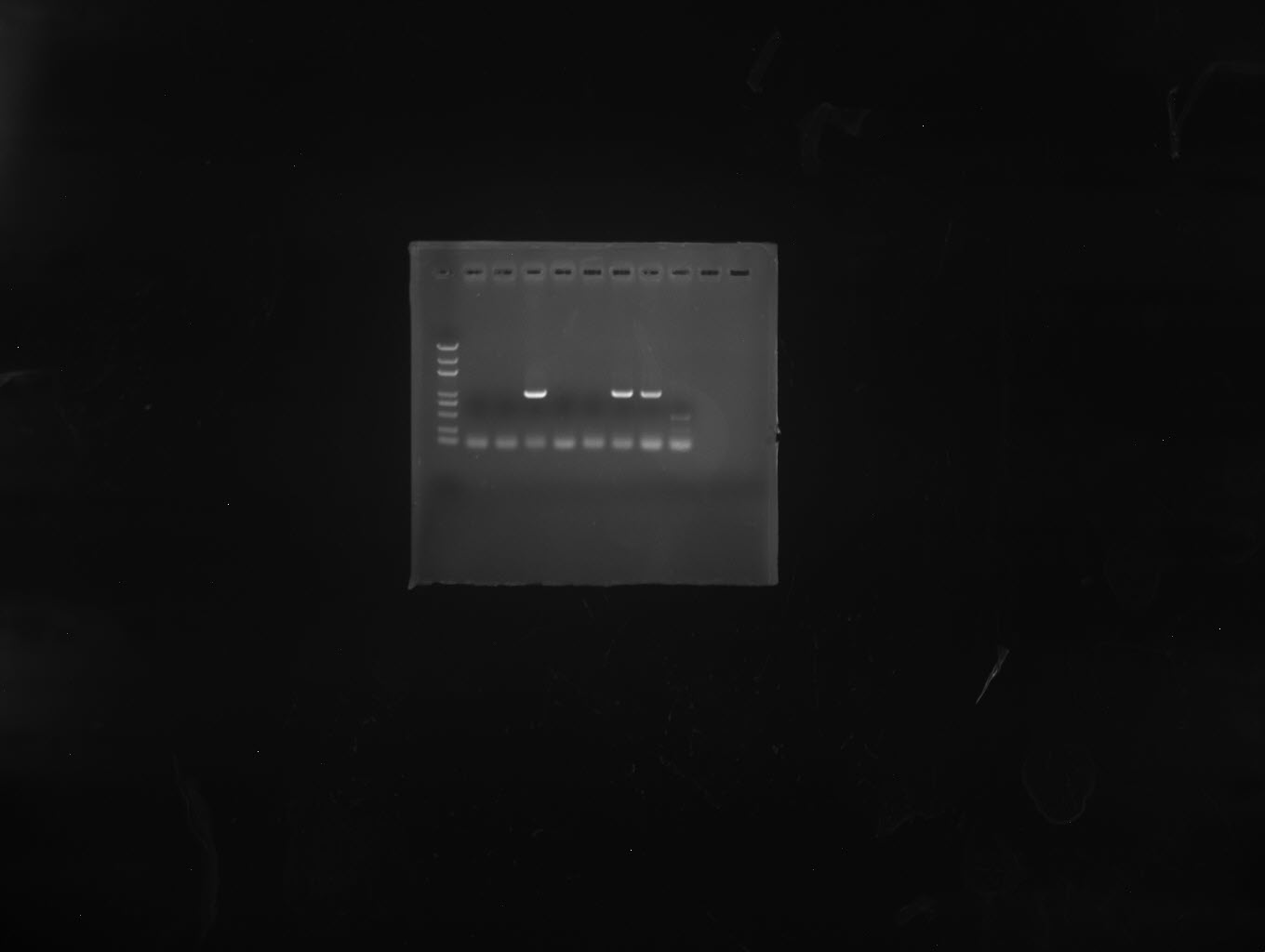

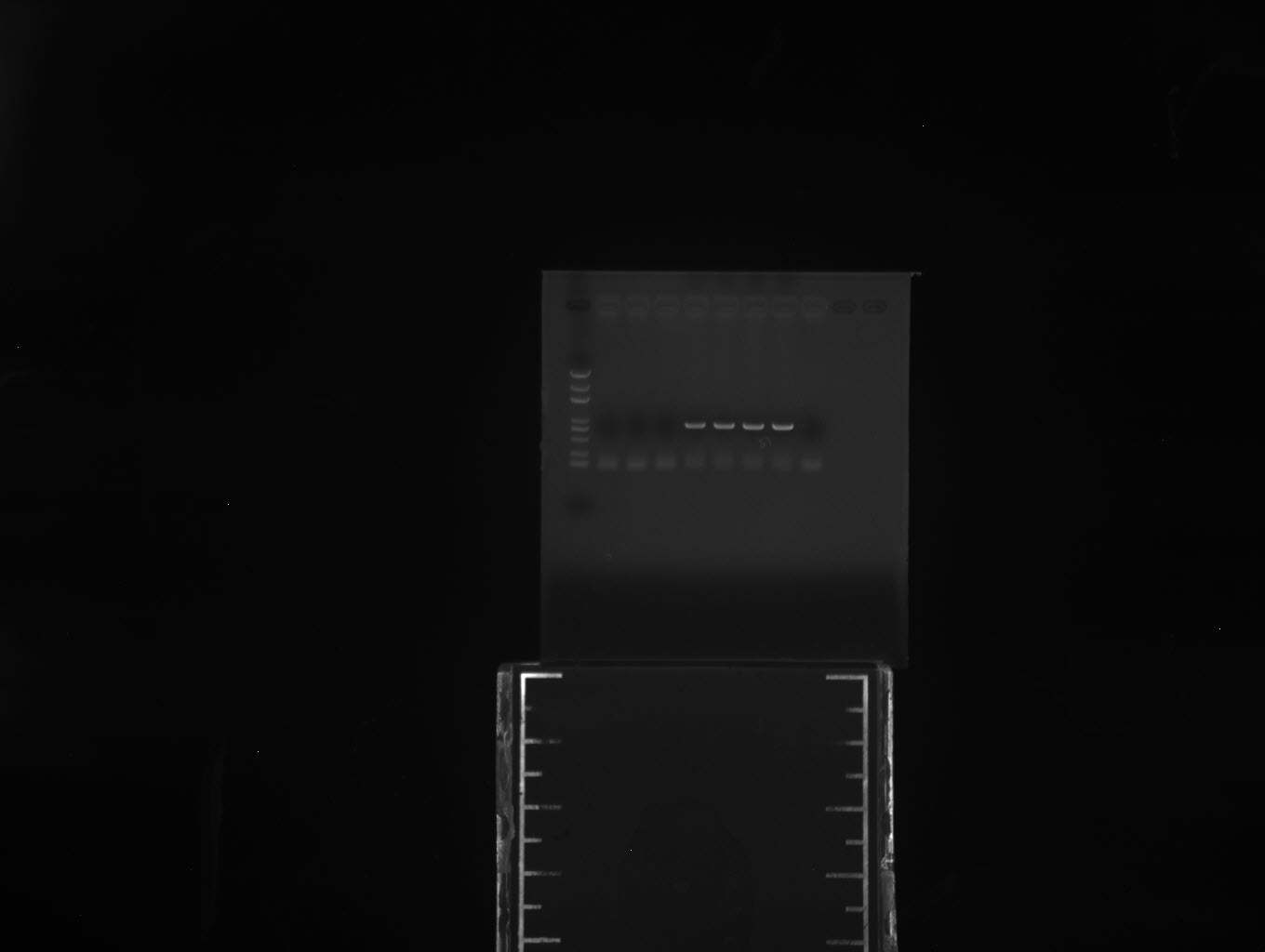
**
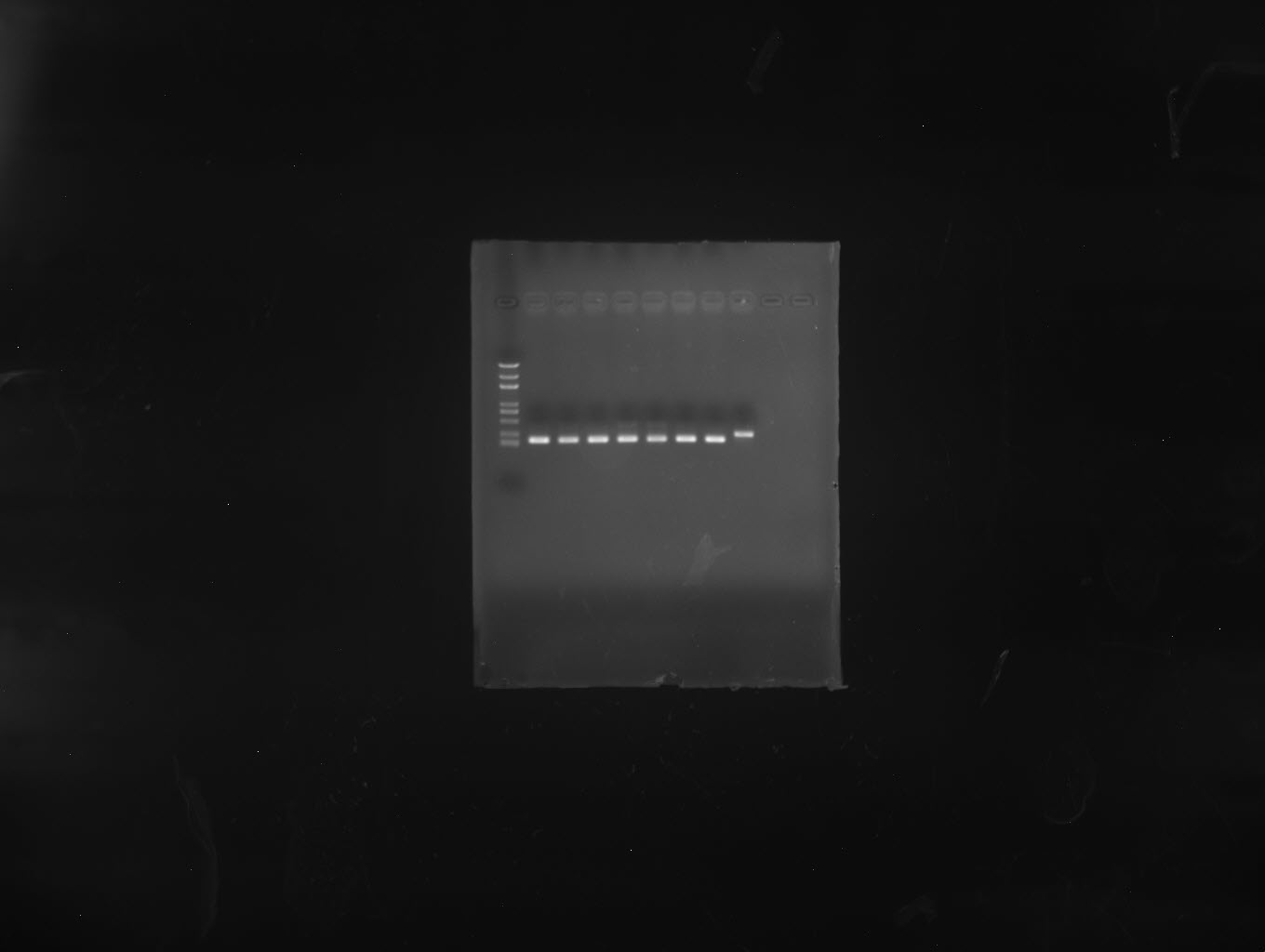


**Figure S1. Detection of the expression levels of the transgenes in *Arabidopsis* leaves by RT-PCR.** The genes used for detection are *CsF3'5'H*, *ANR*, *PAP1* and *PP2A* from left to right. Sample names for each lane are (from left to right): 1. molecular marker; 2. *CsF3’5’H*-over-expression line; 3. *CsANR2*-over-expression line; 4. *pap1-*D mutant line; 5. *CsF3’5’H* × *pap1*-D (F × p) double crossing line; 6. *CsANR2* × *pap1*-D (A × p) double crossing line; 7. *CsF3’5’H* × *CsANR2* × *pap1*-D (F × A × p) triple crossing line. 8. The wild type line as control.
